# Insights of Endocytosis Signaling in Health and Disease

**DOI:** 10.3390/ijms24032971

**Published:** 2023-02-03

**Authors:** Chandramani Pathak, Foram U. Vaidya, Bhargav N. Waghela, Pradip Kumar Jaiswara, Vishal Kumar Gupta, Ajay Kumar, Barani Kumar Rajendran, Kishu Ranjan

**Affiliations:** 1 School of Biomedical Sciences, MGM Institute of Health Sciences, Navi Mumbai 410 209, Maharashtra, India; 2 Gujarat Biotechnology Research Centre, Gandhinagar 382011, Gujarat, India; 3 Department of Microbiology, Atmiya University, Rajkot 360005, Gujarat, India; 4 Department of Zoology, Banaras Hindu University, Varanasi 221005, Uttar Pradesh, India; 5 Department of Pathology, School of Medicine, Yale University, New Haven, CT 06511, USA

**Keywords:** endocytosis, phagocytosis, pinocytosis, receptor endocytosis signaling, chronic diseases

## Abstract

Endocytosis in mammalian cells is a fundamental cellular machinery that regulates vital physiological processes, such as the absorption of metabolites, release of neurotransmitters, uptake of hormone cellular defense, and delivery of biomolecules across the plasma membrane. A remarkable characteristic of the endocytic machinery is the sequential assembly of the complex proteins at the plasma membrane, followed by internalization and fusion of various biomolecules to different cellular compartments. In all eukaryotic cells, functional characterization of endocytic pathways is based on dynamics of the protein complex and signal transduction modules. To coordinate the assembly and functions of the numerous parts of the endocytic machinery, the endocytic proteins interact significantly within and between the modules. Clathrin-dependent and -independent endocytosis, caveolar pathway, and receptor mediated endocytosis have been attributed to a greater variety of physiological and pathophysiological roles such as, autophagy, metabolism, cell division, apoptosis, cellular defense, and intestinal permeabilization. Notably, any defect or alteration in the endocytic machinery results in the development of pathological consequences associated with human diseases such as cancer, cardiovascular diseases, neurological diseases, and inflammatory diseases. In this review, an in-depth endeavor has been made to illustrate the process of endocytosis, and associated mechanisms describing pathological manifestation associated with dysregulated endocytosis machinery.

## 1. Introduction

Endocytosis is a mechanistic process, associated with internalization of the extracellular materials such as microbes, cellular components, nutrients, or macromolecules [1]. Conventionally, eukaryotic cells use the endocytosis process for the absorption of molecules and secretion of signaling transmitters (hormones and cytokines) to maintain cellular homeostasis [2]. Endocytosis machinery is a well-conserved physiological process in lower to higher organisms, which has been frequently acquired for cellular defense, immune responses, uptake, and energy metabolism [3]. Earlier, the endocytosis process was exclusively attributed to the internalization of molecules across the plasma membrane, but with the methodological advancement, it has been found that endocytosis is providing a special route of cellular trafficking and signal transduction [4]. Notably, the cellular communication between the extracellular and intracellular compartment of the cell is becoming incredibly interesting to understand various cellular physiological and developmental processes during normal and pathophysiological consequences [5]. The cell surface receptors have a key role in the recognition of ligands and transduction of signals for various cellular events to define the fate of cells. Many cellular signaling events govern through internalization of plasma membrane receptor endocytosis signaling to coordinate the network between the extracellular and intracellular environment [4]. Christian de Duve in 1963 coined the terminology “endocytosis”, but the concept of the endocytosis process known as phagocytosis was defined by Elie Metchnikoff during the end of the 19th century followed by receiving the Nobel Prize in medicine in 1908. Endocytosis is a well-defined process of cellular logistics for material handling, processing, packaging, and transportation to cellular compartments. 

Mechanistically, there are three types of endocytosis processes described based on the uptake of molecules, characterized as phagocytosis (uptake of large particulate; also considered as the cellular eating process), pinocytosis (uptake of liquid materials or cell drinking), and receptor-mediated endocytosis (considered as a sensor for communications and networking of cellular machinery) [1,2]. The presence of the receptor on the plasma membrane works as a sensor for detecting the signals and transmitting the signal response for the extracellular to intracellular environment of the cells. The receptor-mediated endocytosis has been further classified into several subtypes, such as clathrin-mediated endocytosis (CME), Caveolin-mediated endocytosis (CavME), Caveolin-independent endocytosis (CavIE), and Clathrin-independent endocytosis (CIE) [6,7,8,9]. The endocytic signaling is further regulated and modulated by several intracellular proteins [4]. Endocytosis evolved from prokaryotes to eukaryotes for providing the nutrients and setup the selective communication between extracellular and intracellular compartment of the cell through the plasma membrane. In this review, we systematically present current understanding of the endocytosis process and signaling in human health and disease.

## 2. Types of Endocytosis

### 2.1. Phagocytosis

**Figure 1:**
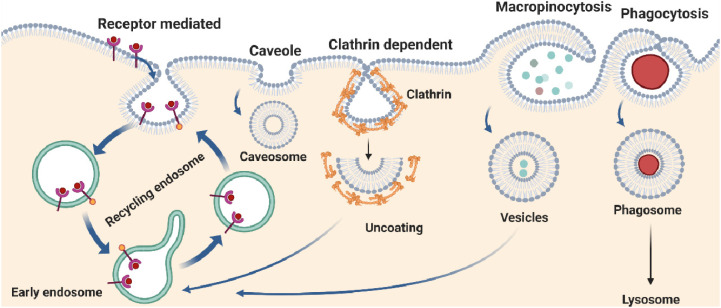
Different pathways of endocytosis. Endocytosis can be broadly classified into pinocytosis and phagocytosis. Pinocytosis involves the internalization of small molecules, whereas phagocytosis involves the internalization of large particles like microbes. Receptor mediated endocytosis is a selective mechanism and the complex proteins recycled to cell membrane. Caveolin encapsulates transported molecules in the caveosomes and protects against lysosomal degradation. Clathrin-mediated endocytosis (CME) requires grafting of specific sorting sequences into membrane downturns and recruits clathrin moieties for cargo internalization.

Phagocytosis is a complex cellular process of multicellular organisms to ingest or engulf solid large particles more than 0.5 µM in size including microorganisms, apoptotic cells, or foreign substances. In unicellular organisms, it is mostly considered as a nutritional support process for survival, and in multicellular organisms it is a vital process involved in regulation of homeostasis pertinent to cellular defense employed by immune cells, including macrophages, monocytes, neutrophils, dendritic, and osteoclasts [10]. In multicellular organisms, phagocytic cells do not unnecessarily attack all types of resident microbes. Interestingly, phagocytic cells are more trained to distinguish symbiotic and pathogenic microbes, and it is therefore sensible to think that phagocytosis machinery is tightly regulated by a highly organized and complex process. Dysfunction of the phagocytosis process has been attributed to accumulate pathogenic loads and leads to hyper immune responses. Mechanistically, phagocytosis consists of several sequential steps: first cellular detection of the target/molecule, followed by internalization and formation of intracellular phagosomes that finally fused with a lysosome to form a phagolysosome (Figure 1) [11]. Membrane dynamics during phagocytosis are critical for the efficient capturing of entities and fusing them with the lysosomes in the cytosol. Notably, the uptake of molecules during phagocytosis is preceded through the receptor selective mechanism, in which receptors form a complex with molecules and enroute to cytoplasm followed by fusion with lysosomes (Figure 1). The phagolysosome is an acidic and hydrolytic formulation, leading to lysis of entities into small antigenic moieties that are transported to the cell membrane and displayed as antigens for immune cells recognition [12]. The process of phagocytosis has been facilitated through Fcγ receptors (FcγRs), scavenger receptors (SRs), and complement receptors (CRs). Importantly, FcγRs and CRs-mediated phagocytosis also contributed to macrophage proliferation and upregulation of proinflammatory TNF-α, IL-1β, IL-6, and MMP-9 cytokine levels in murine macrophages, respectively [13,14], suggesting that distinct phagocytic receptors have a critical role in balancing cellular differentiation and activation during pathogenic encounters. Complement-mediated phagocytosis follows three major pathways: namely alternative, classical, and mannose-binding lectin pathways. Several CRs have been described earlier and annotated based on the functional modalities including, CR1, CR3, and CR4. The CR1 binds with ligand or opsonin C3b, C4b, and C3bi and is responsible for the interaction with microbes or small molecules. Moreover, CR3 and CR4 are integrin family proteins that bind to C3bi and are required for the ingestion of molecules [15]. Notably, complement-mediated phagocytosis may require secondary signals, such as cytokines or chemokines for optimal function, but does not provoke pro-inflammatory mediators and reactive oxygen species (ROS) and is considered as non-immunogenic or anti-inflammatory [10,16]. In contrast, phagocytosis mediated by the FcRs including FcγRI, and FcγRIIA is considered as pro-inflammatory in outcomes. The interaction of the Fc region of antibodies to the Fc receptors leads to tyrosine phosphorylation of ITAMs (immune receptor tyrosine activation motifs) through Src-family kinases such as Syk kinases [17]. The activation of Src/Syk kinases together with PI-3 kinase proteins further induces downstream transcriptional activation of pro-inflammatory cytokines [18]. These studies provide a basis for future studies to address the CRs and FcγRs-mediated phagocytosis process in the immune cells for effective clearance of pathogens and manage the phenotype of the phagocytic cell to be anti-inflammatory.

Apoptosis signaling is a fundamental mechanism through which unwanted and dying cells are removed from the main body stream through two key pathways: death receptor-mediated and mitochondrial-dependent [19,20]. The apoptotic bodies are further selectively ingested by phagocytic cells to avoid local release of cell contents and related local inflammation [21,22,23]. The phagocyte recognizes “find me” and “eat me” signals (such as lysophosphatidylcholine (LPC) and phosphatidyl serine (PS)) displayed on the plasma membrane of apoptotic cells and transduce a signal to the cell machinery required for engulfment [10]. The phagocytes express two kinds of membrane receptors to recognize those “find me” and “eat me” signals: the opsonic receptors including FcRs and CRs, as discussed above, whereas non-opsonic receptors directly recognize the pathogen with the help of pathogen recognition receptor (PRRs), such as mannose receptor (recognize mannans, dectin-1, dectin-2), lectine-like recognition receptor (recognizes β-glucans and integrin), and scavenger receptor (recognize surface components on bacteria and MINCLE (Macrophage inducible Ca2+-dependent lectin receptor) or DC sign (Dendritic Cell Specific ICAM 3-grabbing non-integrin) receptors). The PRRs can recognize glycolipids and damage-associated molecular patterns (DAMPs) including viruses, fungi, and bacteria in the circulation and directly internalized the moieties [24,25,26]. Although TLRs are also knowns as PRRs, they are not considered as non-opsonic receptors. TLRs do not directly initiate phagocytosis but facilitate it more effectively [27]. It is well-accepted that apoptosis is an evolutionary conserved process and is a very essential mechanism to maintain cellular homeostasis in the multicellular organism. Any defects in phagocytosis results in impaired apoptosis machinery and results in a defective immune response and may cause susceptibility to various diseases including autoimmunity, chronic granulomatous disease, myeloperoxidase (MPO) deficiency, Chediak–Higashi syndrome, lazy leukocyte syndrome, leukocyte adhesion deficiency, Job’s syndrome, and chemotactic disorders [28].

### 2.2. Macropinocytosis

Macropinocytosis is generally considered as “cellular drinking”, which is performed by ripples and cups of the plasma membrane to capture small size particles from the surrounding cellular environment and internalized through miniature vesicles followed by fusion with lysosomes and hydrolyzed into discrete nutrients [29,30]. The exact mechanism defining the processing of pinocytic vesicles and cellular processes is not clearly defined. The construction of a macropinosome at the plasma membrane is a receptor-ligand-independent event and driven by actin polymerization and downstream phosphorylation and dephosphorylation of different signaling molecules [31]. The macropinosomes can trap solutes in the range of 0.2–5 μm in diameter and subsequently undergo homotypic fusion with lysosomes followed by release of to the cellular vicinity [32] (Figure 1). Notably, the phagocytosis process internalizes large size particles, whereas macropinocytosis ingests fluid along with smaller size particles. Macropinocytosis occurs in many cell types, including small intestine microvilli where this process is utilized to absorb nutrients. Further, egg cells also use pinocytosis to obtain nutrients before fertilization. The macropinocytosis process is associated with various cellular processes, such as cell growth, cell proliferation, cell death, endocytosis, exocytosis, phagocytosis, chemotaxis, glycolysis, macrophage activation, and proteolysis. Furthermore, macropinocytosis is a coordinated signaling mechanism. A previous report revealed that membrane ruffling and macropinocytosis are regulated by distinct Ras signal transduction pathways [33]. A balanced macropinocytosis signaling in innate immune cells is critical to perform various defense mechanisms, including continuous monitoring of soluble antigens, pathogen sensing, and ingestion of pathogens [30]. In fact, release of growth factors from macrophages, such as colony-stimulating factor-1 (CSF-1), induces macropinocytosis [34]. Moreover, cytokine C-X-C chemokine motif ligand-12 and components of the bacterial cell wall such as lipopolysaccharide (LPS) can stimulate macropinocytosis process [35].

Dysregulation of the macropinocytosis process further corroborates with many physiological and pathological consequences, including dysregulated immune response, dysbiosis, and chronic human disease [1]. A recent study showed that abrogating the expression of lysosomal glutamine and asparagine transporter *SNAT7* regulates mTORC1-dependent macropinocytosis and pancreatic cancer cell proliferation, suggesting nutrients uptake through macropinocytosis is critical for SNAT7-mTORC1 signaling [36]. An earlier study revealed that ATP can serve as a “drink me” signal and is internalized through P2Y_4_ receptors expressed on microglial cells and induces downstream phosphatidylinositol 3-kinase (PI3K)/Akt downstream signaling, and targeting this signaling mechanism could be of promising therapeutic value for the treatment of Alzheimer’s disease. [37]. These studies shed light on the mechanisms driving macropinocytosis and highlights a critical role for macropinocytosis in membrane dynamics and human chronic diseases. 

### 2.3. Receptor Mediated Endocytosis

Receptor-mediated endocytosis is a key cellular event of vascular trafficking where cargo molecules are internalized through binding to a specific receptor present on the plasma membrane known as a clathrin-coated pit and helps to form a membrane vesicle with the help of clathrin proteins [38,39]. Clathrin was discovered in 1975 by Barbara Pearse, who described its key role in the formation of small vesicles for cellular uptake by the process of endocytosis [40]. Further, it is a highly regulated cellular process for internalization of various macromolecules including viral protein, toxins, metabolites, protein growth factors (EGF, PDGF), and polypeptide hormones, such as insulin and luteinizing hormone [41,42,43]. Mechanistically, receptor-mediated endocytosis further categorized as clathrin-mediated endocytosis (CME), clathrin-independent endocytosis (CIE), caveolin-mediated endocytosis (CavME), and caveolin-independent endocytosis (CavIE). Nicotinic acetylcholine receptor is one of the most important receptors responsible for several toxins induced endocytosis [44]. The endocytic signaling is a tightly regulated process and modulated by several intracellular proteins (Figure 1). 

#### 2.3.1. Clathrin-Mediated Endocytosis

##### Clathrin Dependent Endocytosis

Clathrin-mediated endocytosis (CME) is the major endocytic pathway for the internalization of numerous cargos [6]. Clathrin-mediated endocytosis is a term derived from the clathrin protein, which is a key component of the endocytic process. However, in the formation of clathrin-coated endocytic vesicles, more than fifty other cytosolic proteins are involved [45,46]. Clathrin-mediated endocytosis (CME) progress internalization of molecules by complex process signaling mechanism is coordinated by G-protein and tyrosine kinase receptors and synaptic vesicle reformation as an early endosome [7]. Clathrin-mediated endocytosis is one of the most common pathways targeted for drug delivery systems and nano-conjugates preferably transport into cells within vesicles, referred to as early endosomes followed by fusion with cytoplasmic vesicles and matured into late endosomes [47]. Clathrin is a scaffold protein composed of three heavy and three light chains arranging in a triskelion shape (Figure 1). The clathrin complex formation requires the signaling molecules phosphatidylinositol-4,5-bisphosphate (PIP2) and adaptor proteins (AP-2) [48]. However, clathrin-coated vesicles (CCV) are formed with the help of trans-Golgi apparatus (TGA) and AP-1 [49]. Formation of the clathrin-coated pit requires tri-protein complex BAR (Bin/Amphiphysin/Rvs) and actin filament with subsequent dephosphorylation of PIP2 and GTPase dynamin activity [50]. Clathrin-mediated endocytosis has been critically regulated by multiple steps including initiation, cargo selection, maturation, and fission under vigilance of GTPase dynamin checkpoint activity. Dynamin has key role in the maturation of the clathrin-coated pit and is regulated by GSK3β-mediated phosphorylation and calcium-influx-dependent phosphatase calcineurin activation [51]. A recent report revealed that the clathrin-adaptor proteins such as the heterotetrametric adaptor protein AP2 complex and monomeric adaptors including lymphoid myeloid leukemia protein (CALM, known as PICALM) family and epsins, which are proteins of the clathrin assembly, bind to cargo molecules and lipids in the plasma membrane [52,53,54]. The scaffolds such as epidermal growth factor receptor substrate 15 (EPS15), clathrin, intersectins, and epidermal growth factor receptor substrate 15 like 1 (EPS15R) interact with themselves and with the clathrin adaptors to cluster the coat components together [43,55,56]. The pioneer module, made up of the earliest assembling coat proteins, initiates the endocytic process [39,57]. To produce the actin module, a network of actin filaments polymerizes at the endocytic site once the coat has been assembled. The regulatory components and actin filament network are two divisions of this module. The Wiskott–Aldrich syndrome protein (WASP) family of proteins, which are key activators of myosin motor proteins, actin filament nucleation, and dynamin, are part of the regulatory components [38,41]. The complex of actin-related protein 2 (ARP2), ARP3, and several other actin-binding proteins forms the actin filament network, which is made up of actin filaments that have been nucleated by these proteins [58]. The BAR domain proteins interact with dynamin to mediate scission and define the scission module. Finally, the uncoating module’s proteins localize to the endocytic site to promote the disassembly of the endocytic machinery. These proteins include lipid phosphatases, protein kinases, and chaperones, which may also help to regulate this process [38,59]. Importantly, viral particles take advantage of the endocytic membrane trafficking and prefer clathrin-mediated internalization of genetic contents into the host cells [60,61]. The continual emergence and rapid global circulation of SARS-CoV-2 variants [62,63] suggests a strong adaptability of variants across the landscape and the ability to employ diverse endocytic mechanisms to gain entry into host cells [64,65]. Interfering expression of clathrin heavy chain in HEK293 (stably expressing ACE2), limits SARS-CoV-2 viral infectivity [66]. Current evidence suggests that pharmacological inhibition of endocytic pathways potentially regressed replication of novel SARS-CoV-2 lineage B.1.1.529 (Omicron variant) in VeroE6/TMPRSS2 cell, suggesting entry selectivity of variants is associated with clinical manifestation [67].

##### Clathrin in-Dependent Endocytosis

Clathrin-independent endocytosis (CIE) has been implicated in multiple important cellular functions through mediating the absorption of several receptors, extracellular ligands, and pathogens, including viruses and various life-threatening bacterial toxins. There are three main molecular pathways by which CIE carriers develop. Cargo capture by cytosolic proteins is the primary mechanism used by interleukin 2 receptor (IL-2R) endocytosis and fast endophilin-mediated endocytosis (FEME). The membrane remodeling by acute signaling drives macropinocytosis. Finally, the clustering of extracellular lipid or cargo by glycolipid-lectin (GL-Lect) hypothesis facilitates the uptake of cholera and shiga toxins and receptors by the CLIC/GEEC pathway [68].

### 2.4. Fast Endophilin-Mediated Endocytosis

Fast endophilin-mediated endocytosis (FEME) is the first model demonstrated for clathrin-independent endocytosis, based on the membrane curvature-active BAR (Bin/amphiphysin/Rvs)-domain protein family member endophilin [69]. Numerous plasma membrane proteins, including growth factor receptors, heterotrimeric G-proteins, and IL-2 receptors, use FEME, which is triggered by ligands and preferentially occurs at the leading edge of migrating cells. An earlier study identified that the small GTPase Cdc42 brings the BAR-domain proteins FBP17 and CIP4 to the plasma membrane, which then recruits the phosphatases SHIP2 and lamellipodin to stimulate the local synthesis of PIP2, which enriches endophilin [70].

### 2.5. CLIC/GEEC Endocytosis

Another concept for clathrin-independent endocytosis includes short, often crescent-shaped tubular clathrin-independent carriers (CLICs) [9], which mature into early endocytic compartments (GEECs), enriched with glycosylphosphatidylinositol (GPI)-anchored protein [71]. The actin nucleation factor ARP2/3, the small GTPases Arf1 and CDC42, the GTPase activating factor GRAF1, and the BAR domain protein IRSp53 regulate CLIC/GEEC endocytosis [72,73,74]. For the endogenous cargoes, CLIC/GEEC endocytosis is dynamin-independent, but it is not strictly dynamin-dependent for exogenous cargoes including shiga and cholera toxins [72].

### 2.6. GL-Lect Hypothesis

There is a molecular hypothesis according to which pathogenic or cellular sugar-binding proteins bind with glycolipids and rearrange the glycolipids. This binding promotes the biogenesis of tubular endocytic pits [8,9]. Subsequently, CLICs generate from these pits to facilitate the cellular uptake of cellular proteins (e.g., integrins, CD44, CD59), pathogenic products (e.g., cholera and Shiga toxins), or pathogens (e.g., SV40 virus), which are recruited by the galectins [75,76,77]. These clathrin-independent processes (FEME, CLIC/GEEC, and GL-Lect) are sensitive towards the activity of the actin cytoskeleton and membrane organization into raft-type nanodomains [78].

### 2.7. Ion Channel and Endocytosis

Ion channels (IChs) are transmembrane proteins that facilitate transport of ions across the membranes. Ich function is dependent upon their appropriate location and abundance within the cells, which is regulated by a delicate equilibrium between endocytic, secretory, and degradative pathways [79,80,81]. Ichs are frequently internalised via clathrin-mediated endocytosis (CME), which is an extensively studied endocytic process [82,83]. Numerous molecular determinants involved in the cellular trafficking machinery have been identified in the regulation of ICh dependent endocytosis. In addition, specific stimuli, such as hypokalemia receptors, and some drugs can stimulate the endocytosis of many IChs [84]. The voltage-dependent K+ channels such as Kv7.1 and KATP-sensitive (K_ATP_) undergo CME and are involved in tissue formation and insulin secretion, respectively [80,85]. Moreover, two pore domain K+ channel 3.1 (K_2P_3.1) and nerve growth factor (NGF) promotes the CME [86]. The voltage-gated K+ channel Kv1.2 is negatively regulated by RhoA-dependent GTPase and leads to suppression of endocytosis [87].

Clathrin-independent endocytosis (CIE) is another mode for the internalization of ICh [88]. Numerous IChs are found in lipid rafts and caveolae, and human diseases are associated with their altered spatial distribution [89]. The caveolae of aortic smooth muscle cells are the primary site for the localization of the Kir6.1 channel, the major vascular K_ATP_ isoform. The protein kinase C (PKC)-induced internalization of Kir6.1 is prevented by caveolae disruption with MβCD or caveolin-1 siRNA, which indicates the functional role of caveolae compartmentalization [90]. Furthermore, the ARF6-dependent pathway is the dynamin-independent CIE pathway. Some IChs with acidic clusters are driven to the recycling pathway, which is regulated by ARF6 [91]. Moreover, the massive internalization of IChs is triggered by certain specific insults. In brief, the activation of receptors promotes the posttranslational modification (PTM) of IChs and recruitment of mediators including ubiquitin ligases and β-arrestins, which are the key components of ion channel endocytosis [92,93]. In addition, hypokalemic conditions and several drugs—such as Quinidine, a class I antiarrhythmic drug, and Desipramine, a tricyclic antidepressant—can also trigger the endocytosis of IChs [94,95]. Endocytosis significantly regulates the plasma membrane abundance of IChs, which is important in both health and disease.

## 3. Caveolin Mediated Endocytosis

The caveolar pathway involves caveolae, often known as “little caves,” which are bulb-shaped, 50–60 nm plasma membrane invaginations (Figure 1). The peripheral membrane proteins, known as cavins, and integral membrane proteins, known as caveolins participate in the development of caveolae [96]. Dynamin promotes budding of caveolae [97] while caveolar endocytosis is negatively regulated by EHD2 [98]. The cargos such as folic acid, cholera toxin B, albumin, glycosphingolipid analogs, and viruses such as SV40 and EV1, are internalized and endocytosed via caveolae [99]. Caveolin pathways successfully encapsulate large molecules and prevent lysosomal degradation to retain the functional activity of molecules [100]. 

However, there are several pathways that do not use a caveolin coat and are captured by viruses and bacteria to internalize into the host cell. These pathways are dependent upon various molecules, including DNM2/Dynamin-2, cholesterol, tyrosine kinase, small GTPases, and non-caveolar lipid rafts [89].

## 4. Endocytosis in Health and Disease

### 4.1. Cancer

Endocytosis is a complex cellular event involved in homeostasis and communication to extracellular milieu through internalization of the plasma membrane along with its integral membrane proteins, immunoglobulins, receptors and their ligands, and nutrients. It is a crucial signaling event that plays a key role in cell cycle regulation, mitosis, and apoptosis. Endocytosis is a regulated signaling mechanism and plays a potential role in tumor suppressor pathways. It plays a critical role in signaling through endosomes and rescue degradation of signaling molecules involved in cancer signaling, thus it appears as a potential target in oncogenic pathways. Further, endocytosis is involved in activation of certain cancer receptors such as Epidermal growth factor receptor (EGFR), Transferrin receptor (TfR), and Notch receptor [101]. In addition, in human tumors, altered expression of various endocytic regulatory factors such as clathrin-hc, clathrin- like, *Caveolin-1*, *Nexin-1*, and *Numb* along with driver mutations are crucial for endocytosis [102,103]. Endocytic protein *Numb* governs the level and activity of tumor suppressive protein p53. *Numb* inhibits the degradation of p53 by forming the tricomplex of p53 and Hdm2 where it suppresses the ligase activity of Hdm2 [104]. Thus, perturbation in *Numb* levels may alter the expression of the p53-associated cellular process, such as response to DNA damage, induction of checkpoint, and apoptosis-associated proteins [105]. Furthermore, a remarkable decline in the levels of *Numb* expression has been observed in approximately 50% of breast cancer [104]. The disruption of *Numb* expression might have a severe impact on tumorigenesis. The endocytic activity of *Numb* is associated to Numb–Notch interactions for cell proliferation and differentiation. Importantly, endocytosis plays an indispensable role in the aggressive nature of cancer. Endocytosis appears as an important regulator of tumor metastasis [106]. In cancer, deregulation of several endocytic proteins is involved in migration and invasion. There are some metastatic suppressor genes such as Kisspeptin-1 (KISS1), and metastasis suppressor protein 1 (MTSS1) whose activity depends on alteration in the endocytosis process [106]. Kisspeptin-1 (KISS-1) inhibits cell motility, proliferation, invasion, and metastasis in cancers [107]. However, in breast cancer, it induces invasion. MTSS1 acts as a scaffold protein and inhibits the metastasis in various cancers. However, in head and neck squamous cell carcinoma, a low level of MTSS1 augment the EGF signaling and induce cell proliferation [106]. In contrast, a high level of MTSS1 exhibits a negative influence on EGF signaling and triggers metastasis [108]. 

**Figure 2:**
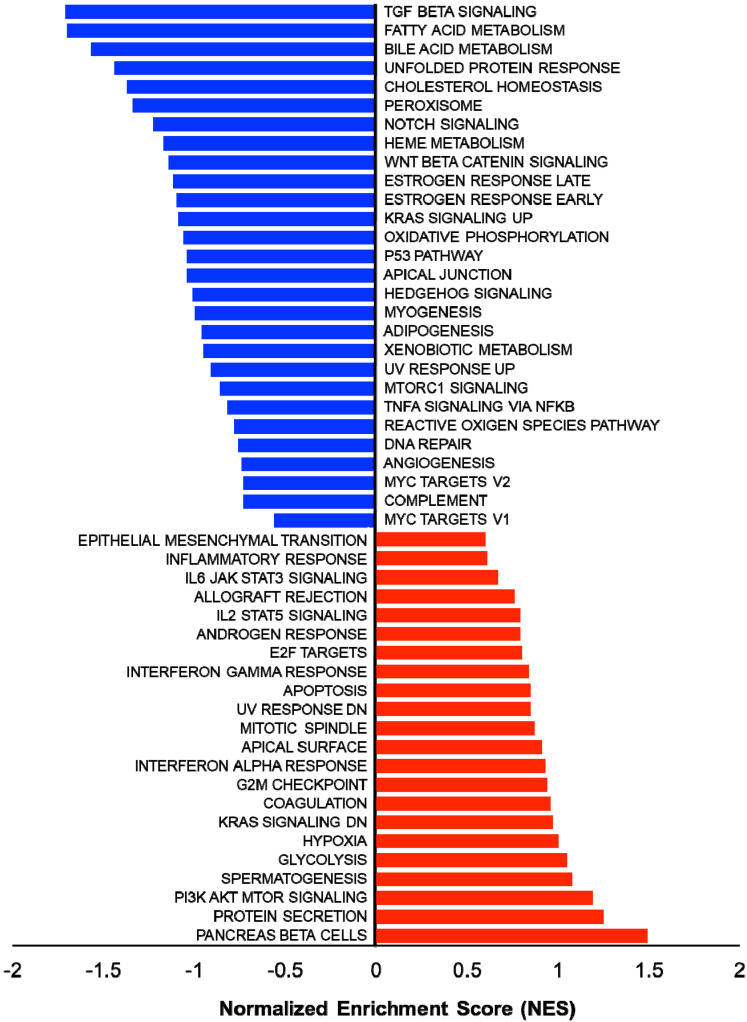
Gene set enrichment analysis (GSEA) showing enriched gene sets of *Rubcn* KO and WT group comparison. Bars in red (positive NES) indicate significant enrichment in KO phenotype and bars in blue indicate WT phenotype (negative NES). Data source available fromGSE118019 [102].

**Figure 3:**
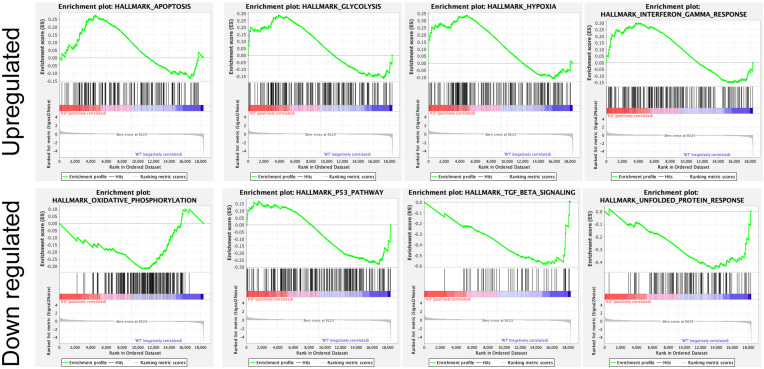
Enrichment plot of top four GSEA hallmarks enriched in *Rubcn* knockout and wildtype. *Rubcn* KO group enriched with significant upregulation in apoptosis, glycolysis, hypoxia, and interferon gamma response gene sets (upper panel). Oxidative phosphorylation, p53, TGFβ signaling, and unfolded protein response enriched in WT group (lower panel).

In addition, several studies showed the involvement of different classes of endocytic proteins in the invasion of multiple cancer types, including colon, breast, colorectal, and non-small cell lung carcinoma (NSCLC). Caveolin endocytic protein shows a regulatory function for breast, prostate, and ovarian cancer. In the early stage of these cancers, caveolin acts as a tumor suppressor, while in advanced stage, it is associated with tumor progression and metastasis [109,110]. Clathrin-mediated endocytic protein AP2 modulates the cell migration and invasion of pancreatic, ovarian, and melanoma cancer through CXCR2 [111]. Endosomal trafficking proteins such as ARF1 regulate breast cancer cell proliferation and migration through regulating the interaction of β1 integrin and protein of focal adhesion (paxillin, Fak, talin) [112,113]. ARF6 promotes cellular motility and invasion of glioma and breast cancer cells by inducing internalization of E-cadherin and breakdown of adherence junction [114]. Further, endocytic proteins of the RAB subfamily such as RAB3C and RAB3D control invasion and metastasis of colorectal and breast cancer, respectively. Elevated expression of RAB3C promotes in vivo migration, invasion, and metastasis of colorectal cancer while RAB3D induced breast cancer cell invasion by activating Akt/GSK-3β/Snail pathway [115,116]. Endosomal-associated protein RAB5 promotes tumor cell migration and invasion, focal adhesion turnover, and integrin trafficking of cancer cells [117,118]. RAB21 controls integrin-mediated cell adhesion and motility of cervical cancer cells. Earlier studies paved a way to identify autophagy signaling proteins as a target to map their interaction with endocytic proteins and cross-regulation in tumor progression. However, identifying such targets is still challenging. Targeting the most viable endocytosis-associated gene(s) may help to achieve this goal. One of the recent studies used RNAseq expression in *Rubcn* knockout (KO) and wildtype (WT) group (GSE118019) [119]. We also analyzed *Rubcn* gene expression in two different conditions to further explore the list of gene signatures, pathways enriched in the presence as well as absence of *Rubcn*. In order to understand the phagocytosis in tumors and their consequences, we performed gene set enrichment analysis (GSEA) (Figure 2). Interestingly, in GSEA, we identified several important pathways such as apoptosis, glycolysis, hypoxia, and IFNγ response enriched in KO group (Figure 3). Similarly, using differential gene expression analysis, we identified several important genes such as *Rab4a*, *Gzme*, *Glrp1* that were upregulated in *Rubcn* KO group. This analysis suggests that the knock-out of endocytosis-associated genes also accelerates a more immunogenic microenvironment. 

Cancer cells are hyper-proliferative and metastasize to the different part of the body. As a result of malignant proliferation, tumor cells need a rapid supply of nutrients for sustained proliferation. Macropinocytosis is a ligand-receptor-independent process and is exploited by cancer cells for rapid nutrient acquisition [120]. An earlier study showed that pancreatic cancer cell line KRPC was able to proliferate in the absence of essential amino acids in the culture media but obtain amino acids through macropinocytosis from extracellularly degraded albumin protein [121]. Macropinocytosis pathway can also facilitate the internalization of essential molecules, such as ATP, to mediate cancer cell proliferation and survival [122]. Macropinocytosis is also involved in K-Ras-mTORC1 signaling and may induce sustained mTORC1 activation and cell proliferation in cancer cells [123]. Moreover, macropinocytosis has been also reported in cell death of glioblastoma cancer cells with constitutive H-Ras activation in glioblastoma cell line U251, resulting in the accumulation macropinosomes and vacuolization of the cells [124].

### 4.2. Cardiovascular Disease

Cardiovascular diseases such as hypertension, coronary heart disease, stroke, and heart failure are the leading causes of mortality and morbidity [125]. Several endocytic proteins including sorting nexin (SNX), epsins, and disabled homolog 2 (Dab2) play an indispensable role in cardiovascular diseases [126]. SNX is a group of cytoplasmic and membrane-associated phosphoinositide binding proteins that play a role in protein trafficking [127,128]. Impairment of SNX pathway is responsible for the development of various forms of cardiovascular disease (CVD) [126]. In addition, SNX gene variants are also linked to CVD. Accumulating reports revealed that SNX exhibits its function by regulating expression and function of G protein-coupled receptors (GPCRs) such as receptor tyrosine kinases (RTKs) and dopamine receptors for the maintenance of blood pressure [129,130]. A previous report demonstrates that an impairment in the structure and function of SNX is associated with hypertension. Renal SNX5 expression regulates insulin degradation enzyme (IDE) activity and is associated with blood insulin and glucose levels [131]. Decreased renal expression of *SNX5* expression leads to further elevation of systolic blood pressure and inhibition of sodium excretion [129]. Further, studies have also established the association of other SNX, such as SNX19, with coronary artery disease. However, the mechanistic pathways behind the SNXs-induced coronary artery disease remain enigmatic [126]. Moreover, SNXs may play a key role in coronary artery pathogenesis by regulating lipid metabolism. The influence of SNXs on a lipid level may be because of the interaction of SNX1, SNX2, and SNX4 with leptin receptors [132]. Furthermore, studies also suggested the abnormal expression of SNX leads to heart failure. Endogenous SNX13 level reduced in failing the heart of mice and human. In *SNX13*-deficient zebra fish, decreased cardiac systolic function was associated with cardiomyocyte apoptosis, and an inhibition of which improves the cardiac dysfunction [133]. In cardiovascular disease, atherosclerosis is a crucial player associated to morbidity and mortality. Epsins are endocytic adaptor proteins associated with cardiovascular disease [134]. Epsins are involved in endothelial cell dysfunction as well initiation and progression of arthrosclerosis through interaction with inositol 1,4,5 triphosphate receptor type 1 (IP3R1) [134]. Furthermore, Dab2, a multifactorial protein, plays a vital role in several cellular functions, including cell adhesion, cell signaling, and endocytosis. More importantly, Dab2 is associated with cholesterol metabolism and low-density lipoprotein (LDL) uptake by regulating the LDL receptor endocytosis. An earlier study suggests that deletion of *Dab2* in liver endothelial cells results in an elevated level of serum LDL and cholesterol [135]. An earlier study shown that the *Dab2* gene variant is associated with increased risk of coronary artery disease [136]. Interestingly, another report showed that quercetin-mediated up-regulation of Dab2 expression attenuates the atherosclerosis [137]. Hence, Dab2 is considered as a new anti-atherosclerosis therapeutic. 

Earlier reports suggested that the CD36 (cluster of differentiation 36), a transmembrane glycoprotein receptor, plays an important role in athero-thrombotic activity and promotes the pathological conditions such as atherosclerosis and thrombosis [138]. CD36 is a pattern recognition receptor (PRR) and multi-functional protein that is majorly involved in the uptake of fatty acids (FA) in adipose tissues and plays a key role in regulation of lipid metabolism [139]. The process of FA uptake and its delivery is facilitated by caveolae-dependent internalization of CD36. Additionally, the FA uptake mediated by CD36 is a palmitoylation-regulated endocytic pathway [140]. The CD36 is expressed on the surface of various cell types including skeletal and cardiac myocytes [141]. It acts as a key player in energetics of cardiac myocytes as it facilitates FA transport, which further utilizes beta oxidation and leads to energy generation. Apart from FA, CD36 also recognizes and interacts with oxidized LDL (oxLDL), which eventually progresses atherosclerosis [142]. The oxLDL has been shown to promote apoptosis signaling in the vascular smooth muscle cells and contributes to atherosclerotic plaques [143]. The expression of CD36 on macrophages and platelets also promotes signaling cascades of inflammation which eventually participates in atherosclerotic arterial lesion formation and thrombus formation [144]. Collectively, CD36 dysfunction has been shown to contribute to the pathologies of atherosclerosis. Therefore, targeting the CD36-mediated transport of lipid moieties could be an effective therapeutic approach for the treatment of atherosclerosis and thrombosis.

Moreover, the intracellular compartmentalization of G protein-coupled receptor (GPCR) during early endosome and Golgi apparatus distribution are associated with cardiovascular outcomes. Endosomal G protein signaling by vasopressin type 2 receptor (V_2_R) plays a key role in cardiac arrest. There are three types of vasopressin receptors, including V1AR, V1BR, and V2R, which are triggered by arginine vasopressin (AVP). An elevated level of AVP plays a crucial role in changing the cardiovascular function and impaired renal solute-free water excretion result in hyponatremia [145,146]. Therefore, vasopressin receptors, V2R, have emerged as a popular target to develop the antagonist against therapeutics for cardiac arrest and hyponatremia. 

### 4.3. Neurological Disorders

Millions of neurons organize and perform the regular functioning of the brain and nervous system. The fundamental role of neurons includes the transmission and receiving of the information or signals and behaving as a unified structure. This communication of information among the neurons is possible due to the presence of junction-like structures, known as synapses. The communication among neurons occurs by electrical or chemical signals. The electrical signal relies on the phenomena known as action potential. The chemical signals are generated through the transmission of various chemicals among neurons. Broadly, these chemicals are defined as neurotransmitters. The neurotransmitters are stored inside the vesicle structures and tend to release at the synaptic cleft of the synapse for the transmission of the information [147]. At the synapse, the release of neurotransmitters relies on the two fundamental biological events, such as exocytosis and endocytosis. The process of exocytosis is responsible for the release of neurotransmitters, while the process of endocytosis is responsible for the recycling of the synaptic vesicle membranes [148]. 

Several events occur during the process of chemical neurotransmission, such as formation of synaptic vesicles (SV), fusion with plasma membrane for releasing neurotransmitters, and recycling of synaptic vesicles. The recycling of synaptic vesicles is the hallmark event which comprises three key steps at the synapse; (i) release of neurotransmitters, (ii) clathrin-mediated endocytosis, and (iii) ultrafast endocytosis [149]. Apart from those mechanisms, several other mechanisms, such as ultrafast bulk endocytosis and activity-dependent bulk endocytosis, are also responsible for the recycling of the vesicles [150,151]. Numerous proteins play vital roles in the mechanisms of endocytosis, including amphiphysin 1 (AMPH1), endophilin A1, clathrin, dynamin, and synaptojanin 1 (SYNJ1) [149]. Moreover, several regulatory proteins are also involved in the regulation of endocytosis. Collectively, these proteins function in an organized manner to propel various endocytic mechanisms, thus efficiently recycling the SVs, which is crucial for continuous supply of neurotransmitter-filled SVs, performance of sensory functions and maintenance of synaptic physiology [150]. These reports suggest that the process of endocytosis and associated proteins plays a significant role in neurotransmission at synapse and maintain the neural homeostasis. 

Alterations in neuronal homeostasis and poor neuronal function lead to several pathological conditions collectively known as neurodegenerative diseases. In the current scenario, neurodegenerative diseases occur prominently in a large population of the world and pose a socio-economic burden [152]. Extensive studies suggest that the dysfunction of endocytosis signaling at synapse participates in the progression of various neurological disorders such as Alzheimer’s disease (AD), Parkinson’s disease, and Amyotrophic lateral sclerosis (ALS). 

Alzheimer’s disease (AD) is the most prevalent type of neurodegenerative disease [153]. The major pathologies associated with AD include accumulation of amyloid-β (Aβ) plaques and development of neurofibrillary tangles (NFTs) due to hyper-phosphorylated tau protein [154]. Deregulation of endocytic processes such as CME and CIE accumulates the (Aβ) plaques and progresses AD [155]. The genome-wide association studies (GWAS) of AD patients revealed that the deregulated expression of genes *PICALM*, *BIN-1*, and *sorLA*, which are essentially involved in clathrin-mediated endocytosis [156,157]. In addition, the altered expression of Rab5, clathrin, dynamin 2, and PICALM has been found in transgenic Tg2576 mice AD experimental models [153,158]. Further, an elevated expression of caveolin-1 reported in the hippocampus and cortex regions of AD brains [159]. The endocytic signaling is considered as tightly regulated cellular process and vulnerable to hyperphosphorylated tau protein [160]. Apart from endocytic proteins and signaling, Endolysosomal or autophagic abnormalities play essential roles in the progression of AD pathologies [160]. The endocytic signaling is also involved in the internalization of extracellular Aβ that accumulates in endosomes and leads to neuronal toxicity [161]. Earlier evidence suggests that the amyloid precursor protein (APP) (a major component involved in the production of Aβ) could be internalized via CME and CIE pathways. Further, the expression level of endocytic proteins, APP, Tau proteins, and other molecules is varied with the age as well as AD progression. The expression of endocytic proteins such as AP180, caveolin-2, clathrin, dynamin-1, flotillin-2, and Rab-5 has been found significantly elevated, and that change accelerates endocytosis and progresses AD in aged brains [153]. Moreover, elevated tau protein induces microtubule assembly and sequesters free dynamins that impair the endocytosis and subsequently perturb the neurotransmission. The neural cells express N-methyl-D-aspartate (NMDA) receptors which are ionotropic glutamate receptors and regulate transmission of glutamate neurotransmitters. The surface expression of NMDA receptors is tightly regulated through clathrin-dependent endocytosis [162,163] and is shown to be endocytosed both in primary neuronal cultures and in vitro heterologous cells [162]. NMDA receptors play an important role in Aβ-induced neurotoxicity [162]. Moreover, Aβ regulates NMDA receptor response by promoting their endocytosis and are associated with synaptic transmission [164]. Therefore, the evidence suggests that the alteration in the endocytosis process and associated protein expression progresses AD.

Parkinson’s disease (PD) is the second most prevalent neurodegenerative disease associated with degeneration and subsequent loss of dopaminergic neurons [165,166]. The synaptic dysfunction is a key event prior to the loss of dopaminergic neurons and participates in the pathogenesis of PD. In a normal human brain, the neurotransmitters release as well as uptake and synaptic vesicles (SV) recycling occur in the synapse. The clathrin-mediated endocytosis participates in SV recycling [151]. Further, the process of synaptic vesicle endocytosis (SVE) regenerates a synaptic vesicle, which is a tightly regulated event and essential for neurotransmission. During PD, the synaptic dysfunction is associated with deregulated SVE signaling. Several genetic studies and mutation analysis suggest that the genes such as *DNAJC6, SYNJ1, SH3GL2, SNCA, LRRK2, PRKN*, and *DJ-1* plays a vital role in the modulation of SVE and progression of PD [149]. The SVE dysfunction leads to erroneous dopamine packaging into the vesicles, as a consequence of elevated cytosolic dopamine and subsequent dopaminergic neurodegeneration [149]. Thus, deregulated expression of endocytic genes and SVE signaling perturb dopamine signaling and subsequent neurotransmission, which promotes the pathologies of PD. α-Synuclein (α-syn) is a 140-amino-acid soluble acidic protein highly expressed in pre-synaptic nerves has been implicated in the pathogenesis of PD [167]. α-syn can regulate clathrin-mediated endocytosis of membrane receptors [168] and is involved in the regulation of NMDA receptor endocytosis [169]. Further studies are needed to explore the mechanistic and therapeutic potential of targeting α-syn and NMDA receptors for the treatment of PD.

Amyotrophic lateral sclerosis (ALS) is a fatal neurodegenerative disease associated with the central nervous system (CNS). The major pathological condition of ALS is the degeneration of the motor neurons in CNS leading to muscles weakness [170]. Occurrence of ALS is sporadic and familial event, and multiple genes are involved in the progression of both types of ALS [171]. The genetic studies revealed that the mutation in the Chromosome 9 open reading frame 72 (*C9ORF72*) progresses ALS. Interestingly, the impaired endocytic signaling has been observed during *C9ORF72* mutated conditions in C9ORF72 ALS/FTD patients as well as the SH-SY5Y cell line model, suggesting that the expression of C9ORF72 modulates endocytosis [172,173]. Further, the expression of C9ORF72 is associated with endocytosis of tropomyosin receptor kinase receptor B (TrkB) (essential for the development and functioning of nervous system) in neurons [170]. Earlier reports suggested that the valosin-containing protein (VCP/p97) regulates endolysosomal sorting of ubiquitylated caveolin-1 [174]. Another protein TDP-43 (nuclear RNA binding protein) inhibits endocytosis and localizes with endocytic proteins in tissue samples of ALS patients. In addition, dynamics of endocytosis modulate TDP-43 expression in a TDP-43 ALS fly model [175].

Collectively, these studies suggest that various endocytic pathways play an essential role in the progression of neurodegenerative diseases. Therefore, targeting the endocytic proteins and signaling mechanism associated with endocytic processes could be the effective approach to target neurological diseases for therapeutic intervention.

### 4.4. Inflammatory Bowel Diseases 

Epithelial integrity and barrier function are critical to separate luminal contents, including nutrients and microbes from the underlying intestinal tissues [176,177]. Perturbations to the epithelial integrity are believed to contribute intestinal dysbiosis and allows heightened microbial penetration, resulting in chronic diseases such as inflammatory bowel disease (IBD), which is an umbrella term for Crohn’s disease (CD) and ulcerative colitis (UC) [178,179]. Endocytotic exodus of microbes in the barrier dysfunction CD, induces mucosal inflammation [180,181]. A case study of a 24-year-old woman with a positive family history of Crohn’s disease showed an increased intestinal permeability precedes the onset of Crohn’s disease [182]. Accumulating evidence shows that the intestinal barrier integrity is chiefly regulated by several multicomplex proteins, constituted of tight junctions (TJs) and adherens junctions (AJs) proteins [183]. Mammalian TJs have diverse roles, ranging from mediating selective diffusion of molecules across the epithelium to cis and trans interactions at sites of intercellular spaces [184]. The claudin protein family of TJs, including junctional adhesion molecule (JAM)-A, the tight junction-associated MARVEL proteins (TAMP), and coxsackievirus and adenovirus receptor (CAR), additionally consist of scaffolding molecules such as the zonula occludens (ZO) protein [185], chiefly regulates the organizational framework of intercellular barrier [186]. These claudin protein family are fundamental to establishing the paracellular passages of nutrients between the intestinal lumen and internal environment, as well as defense mechanisms against pathogens (Table 1) [187,188]. Dysregulated expression of sealing claudins and increased intestinal permeability contributes to a leaky epithelial barrier and may lead to intestinal infection and bowel symptoms of IBD patients [178,189]. The dextran sulfate sodium (DSS) colitis has been described as a most suitable in vivo experimental model of IBD to study intestinal barrier permeability and dissemination of microbes across the intestinal lumen [190,191,192]. Previously in the mouse DSS colitis model, a redistribution of occludin expression was observed compared to distinct appearance at the tight junctions of the apical membrane of colonic epithelium [193]. Apart from claudins, expression levels of other TJ proteins, such as JAM-A, occludin, and ZO-1, remain suppressed during intestinal inflammation [194]. 

Adherens junctions (AJs) are cell–cell adhesion complexes and usually annotated as “cadherins” [195]. Intestinal epithelial cells (IECs) largely express epithelial (E) cadherin, which is required to maintain colonic epithelial barrier permeability, and dysfunction can aggravate colitis [196]. Moreover, dysregulated expression of E-cadherin has been reported in tissue biopsies of IBD patients [197]. 

The proper assembly and functioning of junctional proteins are channelized through exocytic transportation of newly synthesized proteins to the cell surface and recycling of mature TJs and AJs via endocytosis [198]. The defects in TJs and AJs endocytosis leading to barrier disruption in IBD have been reported long ago [199]. The endocytosis of TJs and AJs happens similar to the internalization of luminal antigens into the enterocytes, and IBD patients have an increased ability to transcytosis of luminal antigens [189]. Importantly, recycling and exocytic trafficking of TJs and AJs proteins orchestrated through interaction of carrier vesicles and intracellular organelles [198]. Mechanistically, the membrane-targeted trafficking of carrier vesicles mediated through the protein family of Rab small GTPases and ultimate engrafting to lipid membrane is driven by the SNARE (soluble N-ethylmaleimide-sensitive factor associated receptor) proteins [200]. These results highlight an involvement of organelle-specific trafficking in the establishment of the intestinal epithelial barrier. The intracellular network of endoplasmic reticulum (ER)-Golgi trafficking controls synthesis and recycling of junctional proteins during intestinal inflammation [201]. Notably, the excessive fragmentation and vascularization are the characteristic dysfunction of the ER and the Golgi networks and observed in the intestinal mucosa of UC patients [202].

Altogether, an intact mucosal barrier restricts the infiltration of pathogenic microbes and regulates the absorption and passage of nutrients from the intestinal lumen into the underlying circulation. Endocytosis plays a critical role in the trafficking of intestinal junctional proteins TJs and AJs; however, dysfunction of the junctional proteins is a causative factor in the pathogenesis of IBD. Additional studies may further elucidate the molecular mechanisms associated with the dysfunction of junctional proteins trafficking during intestinal inflammation.

## 5. Conclusions

Endocytosis is a key cellular event that takes place in a sequential manner that regulate the wide range of cellular programming such as apoptosis, opsonization, cell division, cell fate determination, and immune cell functions. Among them, receptor-mediated endocytosis has a vital role in multicellular organisms. The sequential processes of cargo assembly, their transportation, and recognition by target molecules remain tightly regulated to prevent any impairment in normal cellular processes. A balanced cellular coordination of key endocytosis proteins is critical for the proper assembly and transportation of nutrients, microbes, and toxins to maintain cellular homeostasis. However, alteration and dysfunction of this process promotes several chronic diseases, including cancer, neurological disorders such as Alzheimer’s disease, Parkinson’s disease, and amyotrophic lateral sclerosis, cardiovascular disease, and inflammatory bowel disease. Therefore, in-depth scientific validations related to mechanisms and targets of endocytosis pathways may provide a better understanding for the diagnosis and treatment of diseases.

## Figures and Tables

**Table 1:** Expression modulations of claudins in the pathogenesis of IBD.

Claudins	Localization	Role in IBD	CD	UC
Claudin 1	Crypt epithelia	Intestinal barrier permeability	High	High
Claudin 2	Surface colonocytes	Paracellular permeability	High	High
Claudin 3	Crypt epithelia	Intestinal barrier development	Low	Low
Claudin 4	Crypt epithelia	Physiological chloride reabsorption	Low	Low
Claudin 5	Crypt epithelia	Intestinal barrier permeability	Low	ND
Claudin 7	Surface colonocytes	Paracellular flux of small organic solutes	ND	Low
Claudin 8	Crypt epithelia	Na+ reabsorption	Low	ND
Claudin 12	Colonic epithelia	Ca2+ permeability in enterocytes	Low	ND
Claudin 15	Colonic epithelia	Na+ permeability	ND	ND
Claudin 18	Colonic epithelia	Paracellular H+ efflux	ND	High

High, high expression; Low, low expression; ND, not detected.

## Data Availability

Not applicable.
